# Two-sample Mendelian randomization: avoiding the downsides of a powerful, widely applicable but potentially fallible technique

**DOI:** 10.1093/ije/dyx028

**Published:** 2017-03-28

**Authors:** Fernando Pires Hartwig, Neil Martin Davies, Gibran Hemani, George Davey Smith

**Affiliations:** 1 Postgraduate Program in Epidemiology, Federal University of Pelotas, Pelotas, Brazil; 2 Medical Research Council Integrative Epidemiology Unit at the University of Bristol; 3 School of Social and Community Medicine, University of Bristol, Bristol, UK

## Summary data and two-sample Mendelian randomization

Mendelian randomization studies are often performed in an instrumental variables framework, using germline genetic variants as instruments for modifiable disease risk factors or exposures.^1–3^ Mendelian randomization analysis depends on assuming that the genetic variants: (i) are associated with the exposure (the relevance assumption); (ii) have no common cause with the outcome (the independence assumption); and (iii) have effects on the outcome that are solely mediated by the exposure (the exclusion restriction assumption).^1–3^

Summary data Mendelian randomization refers to methods which use summary-level instrument-exposure and instrument-outcome association results (typically, per-allele regression coefficients and standard errors) to obtain causal effect estimates. Two-sample Mendelian randomization refers to the application of Mendelian randomization methods to summary association results estimated in non-overlapping sets of individuals. These data can be obtained from the published literature, typically from summary results provided by consortia of genome-wide association studies (GWAS), or estimated directly from individual-level participant data.^4^ Recent examples include studies evaluating the causal effects of adiposity-related traits on risk of breast, ovarian, prostate, lung and colorectal cancers,^5^ of body mass index on type 2 diabetes^6^ and of telomere length on several health outcomes.^7^ As with Mendelian randomization in general, two-sample Mendelian randomization is analogous to methods originally developed in econometrics.^8^^,^^9^ Recent developments in two-sample Mendelian randomization are based on methods originally developed for meta-analysis.^10^^,^^11^

**Figure 1:**
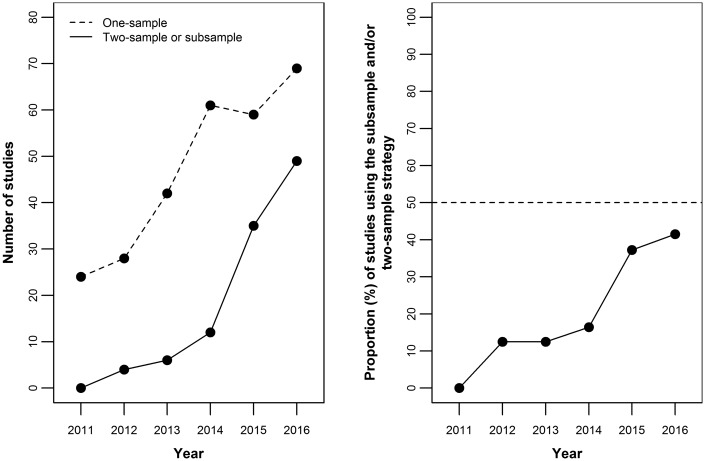
Scatter plots of all empirical Mendelian randomization studies in PubMed from 1 January 2011 to 24 October 2016. Left panel: absolute number of one-sample (dotted line) and subsample and/or two-sample Mendelian randomization studies (solid line). Right panel: proportion of subsample and/or two-sample Mendelian randomization studies (among all one-sample and subsample and/or two-sample studies). The dotted line indicates the 50% value.

Whereas the first formal extended elucidation of Mendelian randomization contained what are in essence two-sample Mendelian randomization estimates (e.g. of the influence of homocysteine on coronary heart disease from partially overlapping meta-analyses of genotype-homocysteine and genotype-coronary heart disease associations),^1^^,^^12^ formal two-sample studies have been a more recent phenomenon. We therefore performed a literature search on 24 October 2016 in PubMed restricted to the period from 1 January 2011 to 24 October 2016 using the terms ‘Mendelian randomisation’ OR ‘Mendelian randomization’ to identify the proportion of Mendelian randomization papers using either the two-sample or subsample^13^ designs. As shown in Figure 1, the proportion rose from 0% in 2011 to 42% in 2016, with a marked increase since 2014. This suggests that two-sample Mendelian randomization already holds a prominent position in the Mendelian randomization literature and its relative importance is likely to continue to grow.

Even though the most natural application of summary data Mendelian randomization methods is in the two-sample setting (i.e. when instrument-exposure and instrument-outcome associations were estimated in non-overlapping sets of individuals), it is possible in principle to use summary data methods in the one-sample context – i.e. when instrument-exposure and instrument-outcome associations are estimated in the same sample. However, summary data Mendelian randomization analyses using instrument-exposure and instrument-outcome associations in the same sample or in partially overlapping samples may be prone to weak instrument bias towards the exposure-outcome estimate that would obtained using conventional methods (typically a regression of the outcome on the exposure). Therefore, using instrument-exposure and instrument-outcome associations estimated in non-overlapping samples is preferable.^14^ However, given that many two-sample Mendelian randomization applications use summary data from large GWAS consortia, it is possible that in many cases the instrument-exposure and instrument-outcome datasets partially overlap due to studies participating in both consortia; and detecting if this occurs and to what degree may depend on careful assessment of the description of the studies included in each consortium. The aims of this paper are to highlight the importance of harmonizing the genetic instrumental variables used for estimating the instrument-exposure and instrument-outcome associations, and to propose steps for doing this and checking the quality of the harmonization process in two-sample Mendelian randomization.

## Guidelines for proper harmonization of datasets for two-sample Mendelian randomization applications

In a paper recently published in the *IJE*, the general issue of data harmonization is discussed.^15^ Appropriate data harmonization is clearly essential when combining two or more independently generated datasets. This is particularly true for two-sample Mendelian randomization, because GWAS results rarely have harmonized effect (or coded) alleles. In genetic association studies, it is often assumed that genetic variants have additive (or per-allele) effects, which corresponds to coding the genotypes numerically according to the number of copies of one of the alleles. So, if a given variant was coded as AA = 0, AC = 1 and CC = 2 (i.e. according to the number of copies of the C allele), then C is termed the effect allele, and A the other (or non-coded, or baseline) allele.

Table 1 provides an overview of the steps typically required to harmonize datasets of summary results of genetic associations for two-sample Mendelian randomization, based on the guidelines provided by Fortier and colleagues.^15^. Below, we will focus on two-sample Mendelian randomization using summary results from GWAS consortia.

**Table 1.** Overview of the data harmonization process for two-sample Mendelian randomization applications, based on the guidelines provided by Fortier and colleagues^15^

Harmonization step	Procedure in two-sample Mendelian randomization
0) Define the research question, objectives and protocol	Prior to data collection, define exposure(s) and outcome(s) variables, data analysis methods, targeted variables, etc. Targeted variables typically include an identifier of the genetic variant, effect and other alleles, effect allele frequency and regression coefficient and standard error
1) Assemble pre-existing data sources and select datasets	Identify potential sources of summary results (e.g. published reports, summary results from GWAS consortia or even individual-level data) and select the most appropriate ones given the research question
2) Evaluate harmonization potential of the selected datasets	At minimum, the effect allele must be available in all datasets to be harmonized. Additional variables, such as the other allele^a^ and effect allele frequency, improve the harmonization potential Missing exposure-associated variants in the variant-outcome dataset may be replaced by proxies available in the latter^b^ but this reduces the quality of the harmonization process iii. Consider whether the populations used to generate the datasets are sufficiently similar to harmonize them
3) Harmonize the data	Identify variants that do not share the same allele pair between datasets, and either correct this if possible^c^ or eliminate such variants. Identify variants with unmatched effect and other alleles and ‘flip’ their effect estimates^d^ and effect allele frequencies^d^ in only one of the datasets
4) Estimate quality of the harmonization process	Strong correlation between effect allele frequencies before and after harmonization, low number of proxy variants used and strong linkage disequilibrium between proxy and index variants suggest good quality of the harmonization process
5) Preserve and disseminate the final harmonized datasets	Publish the harmonized datasets (typically as supplementary material) with all the necessary information to allow replicating the analysis directly from the datasets provided and verifying the quality of the data harmonization process

a Knowing the other allele is particularly useful for harmonization of palindromic variants.

b Variants in high linkage disequilibrium with the index variant in the relevant ancestry group.

c Not having the same allele pair could be a consequence of strand orientation differences between datasets. In this case, harmonizing strand orientation will result in shared allele pairs. Alternatively, if effect allele frequencies are available, they can be used to identify if the effect allele is the major or minor allele, and such classification can be used to check allele matching. Importantly, this strategy would only be reliable if the minor allele frequency is substantially below 50%.

d Multiply by -1 in the case of additive effect estimates (e.g. linear regression coefficients, log(odds ratio), risk differences) or elevate to the power of -1 in the case of multiplicative effect estimates (e.g. odds ratios).

d 1 (or 100%) minus the effect allele frequency in the raw dataset.

Based on the research question, researchers will identify (often multiple) genetic variants associated with an adequate exposure phenotype. For example, if interest lies in studying the causal effects of adiposity measures on a given outcome, then one could use as instruments genetic variants identified in GWAS of anthropometric traits such as body mass index^16^ or waist circumference.^17^ The summary-level association results for the variants that reached genome-wide significance (i.e. *P* < 5.0 × 10^-8^) – hereafter referred to as dataset 1 – are normally extracted from published papers or a web repository.

Although the focus of this paper is on genetic instruments selected based on a GWAS of the exposure phenotype, it is possible that instruments are selected using other criteria. For example, genetic instruments may be selected based on results of functional studies of gene expression regulation or limited to variants located within the gene of interest. For example, the C Reactive Protein (CRP) Coronary Heart Disease (CHD) Genetics Collaboration selected four genetic variants in the *CRP* gene region to evaluate the causal effect of CRP on CHD risk using Mendelian randomization.^18^ These four variants explain 98% of the genetic variation in this locus in populations of European ancestry, and have been shown to regulate circulating CRP levels without changing the protein sequence.^19^ Selecting instruments this way will likely yield results that are less prone to bias due to horizontal pleiotropy compared with selecting genome-wide significant genetic instruments scattered throughout the genome.^20^ In this case, data from the exposure GWAS (e.g. the large CRP GWAS^21^) can be used to obtain precise instrument-exposure (e.g. instrument-CRP) summary association results for the previously chosen instruments (e.g. the four genetic variants in the *CRP* gene region).

**Figure 2:**
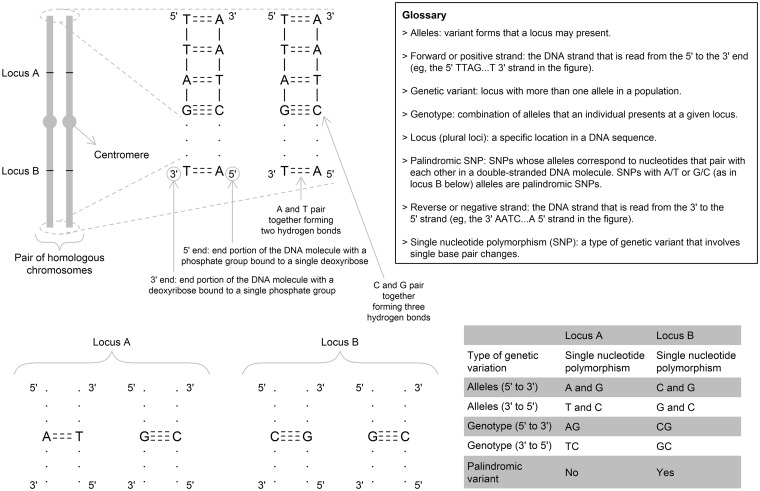
Schematic representation of chromosomes, DNA and genetic variants in a diploid cell.
- Ensure that all variants in dataset 1 are associated with the exposure in the same direction, typically positive (i.e. the exposure-increasing allele is the effect allele). Such standardization is important for two main reasons: (i) it is required when applying a recently-developed summary data Mendelian randomization method called MR-Egger^11^; and (ii) it facilitates interpretation of plots and other forms of presenting results. When a variant is not coded in the desired way, it is necessary to ‘flip’ the variant, which implies that: the effect allele becomes the other allele, and vice versa; the regression coefficient (e.g. ln(odds ratio), mean differences, etc.) must be multiplied by -1; and the effect allele frequency must be subtracted from 1.
- Ensuring that datasets 1 and 2 are identically coded regarding effect (e.g. exposure increasing) and other alleles. For example, if a given instrument has A as the effect allele and C as the other allele in dataset 1, then one must ensure that this same instrument is coded as having A and C as the effect and other alleles, respectively, in dataset 2. Ensuring that both datasets are coded from the same strand is very important to reduce issues with palindromic variants (as discussed in more detail below; see Figure 2). The steps described above are illustrated in Table 2. Notice that, in Table 2, genetic variants are represented by ‘rs’ followed with a number. These are called rs numbers, which uniquely identify a genetic variant and contain information such as location (i.e. chromosome and position on the chromosome), its alleles, and other useful data. Also notice that one of the genetic instruments (rs3) was missing from the outcome GWAS, so it was replaced by an LD proxy (rs5) which was available in both exposure and outcome GWAS. When it is possible to find a suitable LD proxy that is available in both exposure and outcome GWAS, it is preferable to replace the target SNP by its LD proxy in both datasets to avoid problems with phasing (discussed above).

After obtaining dataset 1, the steps listed below are commonly followed:

- Extract – typically from a web repository – summary-level instrument-outcome associations (dataset 2). For example, if the outcome is educational attainment, then one could obtain instrument-outcome associations between each adiposity-associated variant and educational attainment from the educational attainment GWAS.^22^ If a variant in dataset 1 is missing from the outcome GWAS, it can be replaced by a proxy variant in high linkage disequilibrium (LD – association between alleles at two distinct loci within the population; see Figure 2) with it. Ideally, the effect allele used by the LD proxy would be in phase (i.e. located on the same chromosome within a pair of homologous chromosomes) with the required allele for the target single nucleotide polymorphism (SNP). Typical measures of LD (e.g. *r*² or *D*’) are not informative regarding the direction of the correlation between the effect alleles of the target and LD proxy variants. Measures of correlation that are informative of direction (and therefore of phasing), such as Pearson’s correlation, can be estimated for example using publicly available reference panels, such as data from the 1000 Genomes Project for the relevant ancestry group. Of course, a strong positive correlation that is less than 1 (i.e. partial LD) only implies that the effect alleles of the target and the LD proxy variants are typically in phase, but not always (due to recombination events).

**Table 2.** Illustration of the process of data harmonization in two-sample Mendelian randomization using fictional data. Dataset 1 corresponds to instrument-exposure associations, and dataset 2 corresponds to instrument-outcome associations. It is assumed that both datasets are coded in the forward (5’→3’) strand

Step	SNP	Dataset 1				Dataset 2			
		EA	OA	Beta (SE)	EAF (%)	EA	OA	Beta (SE)	EAF (%)
Obtain the raw data	rs1	A	C	−0.1 (0.04)	20	A	C	−0.2 (0.04)	18
	rs2	G	T	−0.2 (0.03)	40	T	G	0.4 (0.03)	58
	rs3^a^	T	C	0.2 (0.03)	60	NA	NA	NA	NA
	rs4	G	A	0.1 (0.04)	80	A	G	−0.2 (0.04)	18
Identify LD proxies	rs1	A	C	−0.1 (0.04)	20	A	C	−0.2 (0.04)	18
	rs2	G	T	−0.2 (0.03)	40	T	G	0.4 (0.03)	58
	rs5^a^	A	G	0.18 (0.03)	58	A	G	0.36 (0.03)	62
	rs4	G	A	0.1 (0.04)	80	A	G	−0.2 (0.04)	18
Standardize the direction in dataset 1	rs1	C	A	0.1 (0.04)	80	A	C	−0.2 (0.04)	18
	rs2	T	G	0.2 (0.03)	60	T	G	0.4 (0.03)	58
	rs5	A	G	0.18 (0.03)	58	A	G	0.36 (0.03)	62
	rs4	G	A	0.1 (0.04)	80	A	G	−0.2 (0.04)	18
Match the alleles in dataset 2 with those in dataset 1	rs1	C	A	0.1 (0.04)	80	C	A	0.2 (0.04)	82
	rs2	T	G	0.2 (0.03)	60	T	G	0.4 (0.03)	58
	rs5	A	G	0.18 (0.03)	58	A	G	0.36 (0.03)	62
	rs4	G	A	0.1 (0.04)	80	G	A	0.2 (0.04)	82

The genetic instrument rs3 was not available in the outcome GWAS. Therefore it was replaced by rs5 which was available in both exposure and outcome GWAS. To this end, rs5 must be in high LD with rs3 in the relevant ancestry group.

LD, linkage disequilibrium; SNP, single nucleotide polymorphism; EA, effect allele; OA, other allele; EAF, effect allele frequency; NA, not available.

Depending on the situation, additional steps may be required. For example, if instruments have been identified from elsewhere (as illustrated above with the CRP example) and an exposure GWAS is only being used to obtain precise estimates of the instrument-exposure association, it is possible that the genetic instruments are missing from the exposure GWAS, and it may be necessary to replace them with LD proxies. Another step, related to but not actually part of the data harmonization process per se, is to adjust the scale of the causal effect estimate. For example, Ference and colleagues wanted to evaluate the causal effect of a 10-mmHg reduction in systolic blood pressure (exposure) on CHD (outcome).^23^ If, for example, instrument-exposure associations (dataset 1) are mmHg changes in systolic blood pressure per copy of the effect allele, then it would be necessary to divide both the regression coefficients and standard errors in dataset 1 (but not in dataset 2) by 10. However, it is important to consider that causal effect estimates from Mendelian randomization may not resemble the estimates from a randomized controlled trial even after scale adjustment, because the genetic variants relate to life-time differences in exposure, whereas in trials the intervention is generally for a much shorter period. Moreover, genetic variants and interventions may affect the exposure through different mechanisms.^24^^,^^25^

The final step would be to undertake quality control checks. One useful check is to compare (either visually or through statistics such as the correlation coefficient, or both) datasets 1 and 2, before and after harmonization, regarding effect allele frequency. If case-control data were used to generate the dataset, it is preferable to use effect allele frequencies estimated in controls only. In Table 2, the Pearson correlation coefficient was -0.003 before harmonization (after identifying LD proxies), and 0.98 after harmonization. A similar check may be applied to standard errors, although linear regression may be preferred over a correlation coefficient because there may be a systematic difference in standard errors if the sample sizes used to generate datasets 1 and 2 are considerably different. Other quality control steps include re-checking that instrument-exposure associations are all coded in the desired direction and that the effect and other alleles are indeed the same between datasets. It is also possible to identify typographical errors by comparing pre- and post-harmonization datasets regarding standard errors, absolute value of the regression coefficients, and minor allele (i.e. the least common allele of a given genetic variant; this may or may not be the effect allele) frequency.

## Potential issues in data harmonization for two-sample Mendelian randomization applications

There might be problems in each step of the data harmonization process. These include: lack of standardization of the direction of the instrument-exposure association; selection of poor LD proxies for missing variants; using negatively, rather than positively, correlated alleles as correspondent between the target SNP and the LD proxy (relevant when the target SNP is used in one of the datasets and the proxy is used in the other dataset, as discussed above); lack of allele harmonization between datasets; different genetic variants (excluding the case of intentionally selected LD proxies) between datasets (which can happen, for example, if variants are labelled as ‘chromosome:position' – e.g. chr1:13960678 – and there is more than one variant mapped to this location); and typographical errors introduced along the process (especially if harmonization is done manually rather than through automated scripts).

Another potential problem in data harmonization is strand issues – i.e. when the effect and other alleles in dataset 1 were defined based on the forward (5’→3’) DNA strand, but on the reverse (3’→5’) strand in dataset 2, or vice versa (Figure 2). Therefore, different studies might report effects of the same SNP using different strands: for example, an SNP with A/G alleles in dataset 1 may be reported as T/C in dataset 2. In most cases these can be identified easily, but palindromic SNPs (i.e. SNPs whose alleles correspond to nucleotides that pair with each other in a DNA molecule; see Figure 2) are much harder to harmonize because the alleles are the same on both strands. These SNPs require that the effect allele frequency is reported, and that the minor allele frequency is substantially below 50% in order to identify ambiguities. There are some options to deal with palindromic SNPs that have minor allele frequencies close to 50%, including: replacing them by suitable, non-palindromic LD proxies; conducting sensitivity analyses to evaluate their impact on Mendelian randomization results; or discarding them.

It is increasingly common in GWAS to perform imputation (i.e. prediction of genotypes of non-genotyped genetic variants in the individuals under study) using genetic datasets (e.g. HapMap^26^ and 1000 Genomes^27^ projects) from more densely genotyped samples as a reference panel.^28^ Therefore, in recent GWAS reports the summary results made available are almost always in reference to the forward strand (Figure 2) as a consequence of imputation to a common reference panel. However, this is not a guarantee that all datasets are ready-harmonized for analyses because different studies may be imputed to different reference panels, or different versions of the same reference panel, which may present differences regarding strand orientation or allele coding.

To illustrate the implications of harmonization issues, consider that we are interested in a single genetic instrument with alleles A and C, so an individual’s genotype for this variant can be AA, AC or CC. Consider that the data are coded as AA = 0, AC = 1 and CC = 2 (i.e. C is the effect allele, and A the other allele). Since choosing the effect allele is often an arbitrary decision, it is possible that effect allele coding differs between instrument-exposure and instrument-outcome datasets. Let us assume that this happened to our genetic variant of interest, so that the instrument-exposure association was estimated with C as the effect allele, but A was the effect allele in the instrument-outcome association. Let ${\hat{\beta }}_{X}$ denote the per-C allele effect estimate of the genetic instrument on the exposure, and ${\hat{\beta }}_{Y}$ denote the per-C allele effect estimate on outcome. In this case, the corresponding per-A allele estimates would be $-{\hat{\beta }}_{X}$ and $-{\hat{\beta }}_{Y}$ (i.e. the per-C allele estimates in opposite direction), respectively. If the allele mismatch is not detected, the causal effect estimated using the ratio method^3^ would be $\left({\hat{\beta }}_{Y}/{-\hat{\beta }}_{X}\right)=-\left({\hat{\beta }}_{Y}/{\hat{\beta }}_{X}\right)$. However, if the alleles are correctly matched, the causal effect estimate would be ${\hat{\beta }}_{Y}/{\hat{\beta }}_{X}$. This illustrates that allele mismatches in a given instrument change the direction of its causal effect estimate, thus reinforcing the importance of allele harmonization in summary data Mendelian randomization.

## Inappropriate data harmonization can distort two-sample Mendelian randomization analysis

Two recently published two-sample Mendelian randomization studies that evaluated the causal effect of CRP levels (exposure) on schizophrenia risk (outcome)^29^^,^^30^ can be used to illustrate the practical relevance of data harmonization for two-sample Mendelian randomization. In both studies, 15 independent CRP-associated variants were used as genetic instruments. These variants were associated with CRP (*P* < 5.0 × 10^-8^) in a GWAS on more than 80 000 individuals,^21^ from which instrument-CRP associations were obtained. Instrument-schizophrenia associations were obtained from the schizophrenia GWAS conducted by the Psychiatric Genomics Consortium (PGC).^31^ Using a method that approximates regressing the outcome on an additive weighted allele score (described in detail elsewhere),^32^ Prins and colleagues detected an odds ratio of 0.86 [95% confidence interval (CI): 0.79; 0.94] per 1-unit increment in ln(CRP) (although they incorrectly interpreted their result as corresponding to a 10% increase in CRP levels).^30^ However, Inoshita and colleagues reported an odds ratio of 1.10 (95% CI: 1.02; 1.19) per 1-unit increment in ln(CRP) when combining multiple instruments using random effects meta-analysis.^29^ Since the same datasets were used, it was possible that these inconsistencies were due to differences in data harmonization.

To investigate this, we extracted regression coefficients, standard errors and effect alleles of all 18 CRP-associated single nucleotide polymorphisms (SNPs) identified in the CRP GWAS from the paper (i.e. dataset 1).^21^ Since only the CRP-increasing alleles were provided in the publication, the other alleles were obtained using the 1000 Genomes browser [browser.1000genomes.org]. None of these variants were palindromic. Summary results for all genetic variants tested in the schizophrenia GWAS were downloaded from the PGC website [https://www.med.unc.edu/pgc/files/resultfiles/scz2.snp.results.txt.gz]. This dataset was reduced to only the 18 variants associated with CRP (*P* < 5.0 × 10^-8^) (dataset 2). Both datasets were sorted according to rs numbers. All variants were identified as having the same allele pairs between datasets. Finally, we checked whether the effect (in this case, CRP-increasing) alleles matched in datasets 1 and 2. They did not: 11 out of 18 variants had different effect alleles. We harmonized the effect alleles in the two datasets, (and the corresponding regression coefficients) using the CRP-increasing alleles as the reference. Applying the inverse variance weighting (IVW) method^10^ to these harmonized datasets yielded an odds ratio of 0.87 (95% CI: 0.79; 0.95) per 1-unit increment in ln(CRP) levels, a result that was consistent with Prins’, but not with Inoshita’s, findings.

To explore the reason for these differences, we compared our harmonized datasets with the datasets provided in Prins’ and Inoshita’s publications. In our analyses using the same two datasets as both Inoshita and colleagues and Prins and colleagues, we found that all 18 of the SNPs that were genome-wide associated with CRP (dataset 1) were available in the schizophrenia GWAS (dataset 2), with no need for proxies, so we used all 18 (see previous paragraph). Inoshita and colleagues removed three of these 18 SNPs from their analyses because, although they were genome-wide significant in the combined discovery and replication sample meta-analyses, they were not in the discovery-only meta-analysis, and the authors had a priori decided that they wanted genome-wide significant variants in both discovery and combined samples. Prins and colleagues reported that five of the 18 CRP genome-wide significant SNPs were not in the schizophrenia GWAS dataset (though we find all 18 are); they found proxies (in tight LD) with two of these but did not include the other three. This means that somewhat different SNPs are used in the three analyses (ours, Inoshita’s and Prins’) — see Supplementary Tables 1-3, available as Supplementary data at *IJE* online. However, as can be seen in these Tables, 13 of the SNPs are identical across all three analysis sets, and the two proxies that Prins *et al*. use are attempting to tag the same associations at the SNPs that they proxy for, both of which were included in Inoshita’s analysis. Thus, Inoshita and colleagues and Prins and colleagues are using highly overlapping sets of genetic instrumental variables for CRP. Indeed, our analysis limiting to the SNPs used in the Prins’ and Inoshita’s studies showed that such differences in variants had almost no influence on the results.

## Use of our suggested harmonization quality control checks to explore the different results

The instrument-CRP coefficients and standard errors in our and Inoshita’s dataset 1 were perfectly positively correlated. This indicates that effect and other alleles (not explicitly provided in Inoshita’s publication) were the same. However, when we compared our dataset 2 after harmonization with Inoshita’s, the correlations between the ln(odds ratios) were -0.82, and the correlations between the standard errors were 0.59. We then compared Inoshita’s dataset 2 with the raw schizophrenia dataset from the PGC. In this comparison, the correlation between the ln(odds ratio) was 0.94, indicating that many variants in Inoshita’s dataset 2 were coded as in the raw PGC dataset. This was suggestive of lack of harmonization between Inoshita’s datasets 1 and 2, because (as discussed above) it was necessary to flip some of the variants in the PGC dataset so that the effect allele was the CRP-increasing allele. Moreover, the correlation between standard errors of 0.59, possibly suggests a typographical error in Inoshita’s dataset 2, since the correlation between standard errors should be 1 even if there are issues with allele harmonization. After graphical inspection, we found that the coefficients and standard errors between Inoshita’s and the raw PGC instrument-schizophrenia datasets were identical aside from the single variant rs4129267. This variant had odds ratio [ln(odds ratio) standard error] of 1.026 (0.012) and 0.990 (0.042) per CRP-decreasing allele in the raw PGC and in Inoshita’s datasets (although in Inoshita’s analysis this was assumed to correspond to the CRP-increasing allele), respectively. We do not know why this difference exists; it might be due to a typographical error because even the standard errors were different. Since all other variants in Inoshita’s dataset 2 were coded as in the raw PGC dataset, we assigned to the rs4129267 variant the same effect and other alleles as in the raw PGC dataset.

Therefore, there were two errors in Inoshita’s dataset 2: (i) lack of allele harmonization between datasets 1 and 2, which resulted in some of the effect alleles in dataset 2 not corresponding to CRP-increasing alleles; and (ii) possibly a typographical error regarding variant rs4129267. Applying the IVW method to Inoshita’s datasets, as provided in their publication, yielded an odds ratio of 1.08 (95% CI: 0.96; 1.22) per 1-unit increment in ln(CRP). After excluding the rs4129267 variant, the odds ratio was 1.09 (95% CI: 0.96; 1.23). After proper harmonization (and exclusion of the variant with the typographical error), the odds ratio was 0.87 (95% CI: 0.79; 0.97). We also re-did our analysis using pre-harmonization datasets, and the resulting odds ratio was 1.09 (95% CI: 0.98; 1.22). These results indicate that the differences between our and Inoshita’s results were largely due to lack of allele harmonization between datasets 1 and 2 rather than to differences in methods to compute the causal effect estimate or the typographical error.

None of the issues above were detected in Prins’ dataset 2. When applying the IVW to their pre-harmonization datasets, the odds ratio was 1.10 (95% CI: 0.99; 1.22). When using post-harmonization datasets, the odds ratio was 0.87 (95% CI: 0.79; 0.95) (almost identical to the result they reported). All results are shown in Table 3. In this example, inappropriate data harmonization shifted the direction of the causal effect estimate from protective (as reported by Prins and colleagues and us) to risk-increasing.

**Table 3.** Odds ratio (95% confidence intervals) of schizophrenia per 1-unit increment in ln(C-reactive protein) based on Mendelian randomization analyses using the inverse variance weighting method, unless indicated otherwise

	Hartwig *et al.*	Inoshita *et al.*	Prins *et al.*
As originally presented in publications	NA	1.10 (1.02; 1.19)^a^	0.86 (0.79; 0.94)^b^
Using pre-harmonization datasets^c^	1.09 (0.98; 1.22)	1.08 (0.96; 1.22)	1.10 (0.99; 1.22)
Using post-harmonization datasets^c^	0.87 (0.79; 0.95)	0.87 (0.79; 0.97)	0.87 (0.79; 0.95)

NA, not applicable.

a Results computed using random effects meta-analysis.

b Results computed using a method that approximates regressing the outcome on an additive weighted allele score.

c Datasets were provided in Supplementary Tables 1-3, available as Supplementary data at *IJE* online.

## Conclusions

Effect allele mismatches can lead to bias in the causal effect estimate in the opposite direction. Although this would not be a problem when the true causal effect is zero, the estimates can be biased if the true causal effect is non-zero. The size of this bias will depend on the proportion of effect allele mismatches and their weight in the causal effect estimate. For example, the bias may just attenuate the causal effect estimate when only a few genetic instruments present effect allele mismatches. However, when most of the instruments or the strongest variants are mismatched, the bias may be strong enough to reverse the causal effect estimate (as in Inoshita and colleagues’ analysis).

Data harmonization has been only briefly discussed in Mendelian randomization guidelines published to date.^33^ In some situations this process may not be trivial, because often various assumptions have to be made or reasonable thresholds have to be decided upon that are potentially study-specific. To prevent bias due to data harmonization errors, we recommend that researchers provide the full, harmonized datasets (as well as the original, pre-harmonization datasets) used in two-sample Mendelian randomization analysis. This would allow evaluation of the harmonization process by reviewers and readers, thus avoiding having to assume that harmonization has been conducted appropriately. We also recommend that harmonization is performed using scripts, and that the scripts are made available with the publication to aid reproducibility. We provide a function written in R [www.r-project.org] that can be used to harmonize summary-level datasets of genetic associations as Supplementary material (available as Supplementary data at *IJE* online) and on GitHub [https://github.com/FernandoHartwig/GenEpi_R_Scripts]. There are others scripts available that aid with harmonization and other steps of two-sample Mendelian randomization analyses, such as those in the TwoSampleMR package [https://github.com/MRCIEU/TwoSampleMR], which is used in the recently developed web database and analysis platform called MR-Base [http://www.mrbase.org/].^34^

Providing both the original and harmonized datasets and describing the harmonization process with enough information for reproducibility will likely minimize the chance that errors in data harmonization are distorting two-sample Mendelian randomization findings. The process of data harmonization should be included when publishing or sharing the analysis code. This practice would also assist reviewers (who should ask for and check harmonized datasets) in assessing whether or not the harmonization has been done correctly. Data harmonization is clearly essential for accurate two-sample Mendelian randomization analyses and should be carefully performed and clearly reported.

Two-sample Mendelian randomization studies are analogous to literature-based meta-analyses, in that they can be performed using publicly available data and can be used to produce publishable papers – receiving consequent academic reward in the absence of any primary data collection or indeed expertise in the field under investigation. In the field of meta-analysis, the corruption of the scientific literature that can occur in this situation has been well documented.^35^ It is important that the recent rise of two-sample Mendelian randomization studies shown in Figure 1 does not recapitulate the epidemic of ‘redundant, misleading, and conflicted systematic reviews and meta-analyses’ recently reported by Ioannidis.^36^ The possibility of this happening is prefigured by the title of a recent paper that suggests that combining the two could be even more (academically) rewarding: ‘Adding Mendelian randomization to a meta-analysis: a burgeoning opportunity’.^37^ It is of concern that the recent substantial increase in the publication of two-sample Mendelian randomization studies could result in poor-quality studies using this design becoming more common in the literature, an occurrence that should be resisted by researchers, reviewers and editors.

## Supplementary Data

Supplementary data are available at *IJE* online.

## Funding

The Medical Research Council (MRC) and the University of Bristol fund the MRC Integrative Epidemiology Unit [MC_UU_12013/1, MC_UU_12013/9]. N.M.D. is supported by the Economics and Social Research Council (ESRC) via a Future Research Leaders Fellowship [ES/N000757/1].

## Supplementary Material

Supplementary Data
