# Supplementary material for: Two-sample Mendelian randomization: avoiding the downsides of a powerful, widely applicable but potentially fallible technique
Source: Int J Epidemiol. 2017 Mar 28;45(6):1717–26. doi: 10.1093/ije/dyx028 (PMC5722032; doi:10.1093/ije/dyx028)
Supplement: Supplementary Data [file dyx028_supp.zip › dyx028-suppl_data/C16-64 Hartwig editorial Supplementary Tables.docx]

**Two-sample Mendelian randomisation: avoiding the downsides of a powerful, widely applicable but potentially fallible technique**

Fernando Pires Hartwig^1,2*^, Neil Martin Davies^2,3^, Gibran Hemani^2,3^ and George Davey Smith^2,3^

^1^Postgraduate Program in Epidemiology, Federal University of Pelotas, Pelotas, Brazil.

^2^Medical Research Council Integrative Epidemiology Unit at the University of Bristol, BS8 2BN, United Kingdom.

^3^School of Social and Community Medicine, University of Bristol, Barley House, Oakfield Grove, Bristol, BS8 2BN, United Kingdom.

*Corresponding author. Postgraduate Program in Epidemiology, Federal University of Pelotas, Pelotas (Brazil) 96020-220. Phone: 55 53 81068670. E-mail: [fernandophartwig@gmail.com](mailto:fernandophartwig@gmail.com).

**SUPPLEMENTARY TABLES**

**Supplementary Table 1.** Instrument-CRP summary results (dataset 1) and instrument-schizophrenia summary results (dataset 2) extracted from the CRP GWAS publication and from the PGC website.

| **Stage** | **Dataset 1** |  |  |  |  | **Dataset 2** |  |  |  |  |
| --- | --- | --- | --- | --- | --- | --- | --- | --- | --- | --- |
|  | **SNP** | **A1** | **A2** | **Beta** | **SE** | **SNP** | **A1** | **A2** | **Beta** | **SE** |
| Pre- | rs10521222 | C | T | 0.104 | 0.015 | rs10521222 | T | C | 0.00909848298525935 | 0.0325 |
| harmonisation | rs10745954 | A | G | 0.039 | 0.006 | rs10745954 | A | G | -0.02510244656084140 | 0.0106 |
|  | rs1183910 | G | A | 0.149 | 0.006 | rs1183910 | A | G | 0.02779997485767900 | 0.0112 |
|  | rs12037222 | A | G | 0.045 | 0.007 | rs12037222 | A | G | -0.00950503001430495 | 0.0128 |
|  | rs12239046 | C | T | 0.047 | 0.006 | rs12239046 | T | C | 0.00340419913356236 | 0.0110 |
|  | rs1260326 | T | C | 0.072 | 0.005 | rs1260326 | T | C | -0.00609855846186947 | 0.0108 |
|  | rs13233571 | C | T | 0.054 | 0.009 | rs13233571 | T | C | -0.01309537131224090 | 0.0167 |
|  | rs1800961 | C | T | 0.088 | 0.015 | rs1800961 | T | C | -0.03069634900259760 | 0.0300 |
|  | rs2794520 | C | T | 0.160 | 0.006 | rs2794520 | T | C | 0.02199629787189610 | 0.0110 |
|  | rs2847281 | A | G | 0.031 | 0.006 | rs2847281 | A | G | 0.01129595974185160 | 0.0108 |
|  | rs340029 | T | C | 0.032 | 0.006 | rs340029 | T | C | 0.00430073855169222 | 0.0112 |
|  | rs4129267 | C | T | 0.079 | 0.005 | rs4129267 | T | C | 0.02569698608718600 | 0.0108 |
|  | rs4420065 | C | T | 0.090 | 0.005 | rs4420065 | T | C | 0.02239729744203830 | 0.0110 |
|  | rs4420638 | A | G | 0.236 | 0.009 | rs4420638 | A | G | -0.00859684722551828 | 0.0146 |
|  | rs4705952 | G | A | 0.042 | 0.007 | rs4705952 | A | G | 0.00330453400830047 | 0.0121 |
|  | rs6734238 | G | A | 0.050 | 0.006 | rs6734238 | A | G | 0.00269636154774253 | 0.0110 |
|  | rs6901250 | A | G | 0.035 | 0.006 | rs6901250 | A | G | -0.01329802814398980 | 0.0113 |
|  | rs9987289 | G | A | 0.069 | 0.011 | rs9987289 | A | G | 0.08320079259276070 | 0.0188 |
| Post- | rs10521222 | C | T | 0.104 | 0.015 | rs10521222 | C | T | -0.00909848298525935 | 0.0325 |
| harmonisation | rs10745954 | A | G | 0.039 | 0.006 | rs10745954 | A | G | -0.02510244656084140 | 0.0106 |
|  | rs1183910 | G | A | 0.149 | 0.006 | rs1183910 | G | A | -0.02779997485767900 | 0.0112 |
|  | rs12037222 | A | G | 0.045 | 0.007 | rs12037222 | A | G | -0.00950503001430495 | 0.0128 |
|  | rs12239046 | C | T | 0.047 | 0.006 | rs12239046 | C | T | -0.00340419913356236 | 0.0110 |
|  | rs1260326 | T | C | 0.072 | 0.005 | rs1260326 | T | C | -0.00609855846186947 | 0.0108 |
|  | rs13233571 | C | T | 0.054 | 0.009 | rs13233571 | C | T | 0.01309537131224090 | 0.0167 |
|  | rs1800961 | C | T | 0.088 | 0.015 | rs1800961 | C | T | 0.03069634900259760 | 0.0300 |
|  | rs2794520 | C | T | 0.160 | 0.006 | rs2794520 | C | T | -0.02199629787189610 | 0.0110 |
|  | rs2847281 | A | G | 0.031 | 0.006 | rs2847281 | A | G | 0.01129595974185160 | 0.0108 |
|  | rs340029 | T | C | 0.032 | 0.006 | rs340029 | T | C | 0.00430073855169222 | 0.0112 |
|  | rs4129267 | C | T | 0.079 | 0.005 | rs4129267 | C | T | -0.02569698608718600 | 0.0108 |
|  | rs4420065 | C | T | 0.090 | 0.005 | rs4420065 | C | T | -0.02239729744203830 | 0.0110 |
|  | rs4420638 | A | G | 0.236 | 0.009 | rs4420638 | A | G | -0.00859684722551828 | 0.0146 |
|  | rs4705952 | G | A | 0.042 | 0.007 | rs4705952 | G | A | -0.00330453400830047 | 0.0121 |
|  | rs6734238 | G | A | 0.050 | 0.006 | rs6734238 | G | A | -0.00269636154774253 | 0.0110 |
|  | rs6901250 | A | G | 0.035 | 0.006 | rs6901250 | A | G | -0.01329802814398980 | 0.0113 |
|  | rs9987289 | G | A | 0.069 | 0.011 | rs9987289 | G | A | -0.08320079259276070 | 0.0188 |

SNP: single nucleotide polymorphism. A1: Effect (or coded) allele. A2: other (or non-coded) allele. Beta: per-effect allele changes in ln(CRP) levels (Dataset 1) or in ln(odds ratio) of schizophrenia (Dataset 2). SE: standard error.

**Supplementary Table 2.** Instrument-CRP summary results (dataset 1) and instrument-schizophrenia summary results (dataset 2) extracted from Inoshita and colleagues publication. Alleles were imputed as described in the main text.

| **Stage** | **Dataset 1** |  |  |  |  | **Dataset 2** |  |  |  |  |
| --- | --- | --- | --- | --- | --- | --- | --- | --- | --- | --- |
|  | **SNP** | **A1** | **A2** | **Beta** | **SE** | **SNP** | **A1** | **A2** | **Beta** | **SE** |
| Pre- | rs10521222 | C | T | 0.104 | 0.015 | rs10521222 | T | C | 0.008959741 | 0.033 |
| harmonisation | rs10745954 | A | G | 0.039 | 0.006 | rs10745954 | A | G | -0.025317810 | 0.011 |
|  | rs1183910 | G | A | 0.149 | 0.006 | rs1183910 | A | G | 0.027615170 | 0.011 |
|  | rs12037222 | A | G | 0.045 | 0.007 | rs12037222 | A | G | -0.009040745 | 0.013 |
|  | rs12239046 | C | T | 0.047 | 0.006 | rs12239046 | T | C | 0.002995509 | 0.011 |
|  | rs1260326 | T | C | 0.072 | 0.005 | rs1260326 | T | C | -0.006018072 | 0.011 |
|  | rs13233571 | C | T | 0.054 | 0.009 | rs13233571 | T | C | -0.013085240 | 0.017 |
|  | rs1800961 | C | T | 0.088 | 0.015 | rs1800961 | T | C | -0.030459210 | 0.030 |
|  | rs2794520 | C | T | 0.160 | 0.006 | rs2794520 | T | C | 0.021761490 | 0.011 |
|  | rs340029 | T | C | 0.032 | 0.006 | rs340029 | T | C | 0.003992021 | 0.011 |
|  | rs4129267 | C | T | 0.079 | 0.005 | rs4129267 | T | C | -0.010050340 | 0.042 |
|  | rs4420065 | C | T | 0.090 | 0.005 | rs4420065 | T | C | 0.022739490 | 0.011 |
|  | rs4420638 | A | G | 0.236 | 0.009 | rs4420638 | A | G | -0.009040745 | 0.015 |
|  | rs6734238 | G | A | 0.050 | 0.006 | rs6734238 | A | G | 0.002995509 | 0.011 |
|  | rs9987289 | G | A | 0.069 | 0.011 | rs9987289 | A | G | 0.083421610 | 0.019 |
| Post- | rs10521222 | C | T | 0.104 | 0.015 | rs10521222 | C | T | -0.008959741 | 0.033 |
| harmonisation | rs10745954 | A | G | 0.039 | 0.006 | rs10745954 | A | G | -0.025317810 | 0.011 |
|  | rs1183910 | G | A | 0.149 | 0.006 | rs1183910 | G | A | -0.027615170 | 0.011 |
|  | rs12037222 | A | G | 0.045 | 0.007 | rs12037222 | A | G | -0.009040745 | 0.013 |
|  | rs12239046 | C | T | 0.047 | 0.006 | rs12239046 | C | T | -0.002995509 | 0.011 |
|  | rs1260326 | T | C | 0.072 | 0.005 | rs1260326 | T | C | -0.006018072 | 0.011 |
|  | rs13233571 | C | T | 0.054 | 0.009 | rs13233571 | C | T | 0.013085240 | 0.017 |
|  | rs1800961 | C | T | 0.088 | 0.015 | rs1800961 | C | T | 0.030459210 | 0.030 |
|  | rs2794520 | C | T | 0.160 | 0.006 | rs2794520 | C | T | -0.021761490 | 0.011 |
|  | rs340029 | T | C | 0.032 | 0.006 | rs340029 | T | C | 0.003992021 | 0.011 |
|  | rs4129267 | C | T | 0.079 | 0.005 | rs4129267 | C | T | 0.010050340 | 0.042 |
|  | rs4420065 | C | T | 0.090 | 0.005 | rs4420065 | C | T | -0.022739490 | 0.011 |
|  | rs4420638 | A | G | 0.236 | 0.009 | rs4420638 | A | G | -0.009040745 | 0.015 |
|  | rs6734238 | G | A | 0.050 | 0.006 | rs6734238 | G | A | -0.002995509 | 0.011 |
|  | rs9987289 | G | A | 0.069 | 0.011 | rs9987289 | G | A | -0.083421610 | 0.019 |

SNP: single nucleotide polymorphism. A1: Effect (or coded) allele. A2: other (or non-coded) allele. Beta: per-effect allele changes in ln(CRP) levels (Dataset 1) or in ln(odds ratio) of schizophrenia (Dataset 2). SE: standard error.

**Supplementary Table 3.** Instrument-CRP summary results (dataset 1) and instrument-schizophrenia summary results (dataset 2) extracted from Prins and colleagues publication.

| **Stage** | **Dataset 1** |  |  |  |  | **Dataset 2** |  |  |  |  |
| --- | --- | --- | --- | --- | --- | --- | --- | --- | --- | --- |
|  | **SNP** | **A1** | **A2** | **Beta** | **SE** | **SNP** | **A1** | **A2** | **Beta** | **SE** |
| Pre- | rs10521222 | C | T | 0.104 | 0.015 | rs10521222 | T | C | 0.009 | 0.033 |
| harmonisation | rs10745954 | A | G | 0.039 | 0.006 | rs7972145^a^ | T | C | -0.018 | 0.011 |
|  | rs12037222 | A | G | 0.045 | 0.007 | rs2293476^b^ | C | G | -0.010 | 0.013 |
|  | rs12239046 | C | T | 0.047 | 0.006 | rs12239046 | T | C | 0.003 | 0.011 |
|  | rs1260326 | T | C | 0.072 | 0.005 | rs1260326 | T | C | -0.006 | 0.011 |
|  | rs13233571 | C | T | 0.054 | 0.009 | rs13233571 | T | C | -0.013 | 0.017 |
|  | rs1800961 | C | T | 0.088 | 0.015 | rs1800961 | T | C | -0.031 | 0.030 |
|  | rs2794520 | C | T | 0.16 | 0.006 | rs2794520 | T | C | 0.022 | 0.011 |
|  | rs2847281 | A | G | 0.031 | 0.006 | rs2847281 | A | G | 0.011 | 0.011 |
|  | rs340029 | T | C | 0.032 | 0.006 | rs340029 | T | C | 0.004 | 0.011 |
|  | rs4129267 | C | T | 0.079 | 0.005 | rs4129267 | T | C | 0.026 | 0.011 |
|  | rs4420065 | C | T | 0.09 | 0.005 | rs4420065 | T | C | 0.022 | 0.011 |
|  | rs4705952 | G | A | 0.042 | 0.007 | rs4705952 | A | G | 0.003 | 0.012 |
|  | rs6734238 | G | A | 0.05 | 0.006 | rs6734238 | A | G | 0.003 | 0.011 |
|  | rs6901250 | A | G | 0.035 | 0.006 | rs6901250 | A | G | -0.013 | 0.011 |
|  | rs10521222 | C | T | 0.104 | 0.015 | rs10521222 | T | C | 0.009 | 0.033 |
|  | rs10745954 | A | G | 0.039 | 0.006 | rs7972145 | T | C | -0.018 | 0.011 |
|  | rs12037222 | A | G | 0.045 | 0.007 | rs2293476 | C | G | -0.010 | 0.013 |
| Post- | rs10521222 | C | T | 0.104 | 0.015 | rs10521222 | T | C | -0.009 | 0.033 |
| harmonisation | rs10745954 | A | G | 0.039 | 0.006 | rs7972145^a^ | T | C | -0.018 | 0.011 |
|  | rs12037222 | A | G | 0.045 | 0.007 | rs2293476^b^ | C | G | -0.010 | 0.013 |
|  | rs12239046 | C | T | 0.047 | 0.006 | rs12239046 | T | C | -0.003 | 0.011 |
|  | rs1260326 | T | C | 0.072 | 0.005 | rs1260326 | T | C | -0.006 | 0.011 |
|  | rs13233571 | C | T | 0.054 | 0.009 | rs13233571 | T | C | 0.013 | 0.017 |
|  | rs1800961 | C | T | 0.088 | 0.015 | rs1800961 | T | C | 0.031 | 0.03 |
|  | rs2794520 | C | T | 0.160 | 0.006 | rs2794520 | T | C | -0.022 | 0.011 |
|  | rs2847281 | A | G | 0.031 | 0.006 | rs2847281 | A | G | 0.011 | 0.011 |
|  | rs340029 | T | C | 0.032 | 0.006 | rs340029 | T | C | 0.004 | 0.011 |
|  | rs4129267 | C | T | 0.079 | 0.005 | rs4129267 | T | C | -0.026 | 0.011 |
|  | rs4420065 | C | T | 0.090 | 0.005 | rs4420065 | T | C | -0.022 | 0.011 |
|  | rs4705952 | G | A | 0.042 | 0.007 | rs4705952 | A | G | -0.003 | 0.012 |
|  | rs6734238 | G | A | 0.050 | 0.006 | rs6734238 | A | G | -0.003 | 0.011 |
|  | rs6901250 | A | G | 0.035 | 0.006 | rs6901250 | A | G | -0.013 | 0.011 |
|  | rs10521222 | C | T | 0.104 | 0.015 | rs10521222 | T | C | -0.009 | 0.033 |
|  | rs10745954 | A | G | 0.039 | 0.006 | rs7972145 | T | C | -0.018 | 0.011 |
|  | rs12037222 | A | G | 0.045 | 0.007 | rs2293476 | C | G | -0.010 | 0.013 |

SNP: single nucleotide polymorphism. A1: Effect (or coded) allele. A2: other (or non-coded) allele. Beta: per-effect allele changes in ln(CRP) levels (Dataset 1) or in ln(odds ratio) of schizophrenia (Dataset 2). SE: standard error.

^a^rs7972145 is a proxy of the target SNP rs10745954. The T and C alleles in rs7972145 correspond to the A and G alleles in rs10745954, respectively.

^b^rs2293476 is a proxy of the target SNP rs12037222. The C and G alleles in rs2293476 correspond to the A and G alleles in rs12037222, respectively.
