# Supplementary material for: Two-sample Mendelian randomization: avoiding the downsides of a powerful, widely applicable but potentially fallible technique
Source: Int J Epidemiol. 2017 Mar 28;45(6):1717–26. doi: 10.1093/ije/dyx028 (PMC5722032; doi:10.1093/ije/dyx028)
Supplement: Supplementary Data [file dyx028_supp.zip › dyx028-suppl_data/C16-64 Hartwig editorial Supplementary Material.docx]

**Two-sample Mendelian randomisation: avoiding the downsides of a powerful, widely applicable but potentially fallible technique**

Fernando Pires Hartwig^1,2*^, Neil Martin Davies^2,3^, Gibran Hemani^2,3^ and George Davey Smith^2,3^

^1^Postgraduate Program in Epidemiology, Federal University of Pelotas, Pelotas, Brazil.

^2^Medical Research Council Integrative Epidemiology Unit at the University of Bristol, BS8 2BN, United Kingdom.

^3^School of Social and Community Medicine, University of Bristol, Barley House, Oakfield Grove, Bristol, BS8 2BN, United Kingdom.

*Corresponding author. Postgraduate Program in Epidemiology, Federal University of Pelotas, Pelotas (Brazil) 96020-220. Phone: 55 53 81068670. E-mail: [fernandophartwig@gmail.com](mailto:fernandophartwig@gmail.com).

**SUPPLEMENTARY MATERIAL**

#1) This function is aimed at harmonising two summary-level datasets of genetic associations by:

#1.1) Keeping only variants available in both datasets.

#1.2) Excluding variants that do not have the same allele pair between datasets.

#1.3) Flipping alleles, betas (see 4.1.4) and effect allele frequencies (EAFs) (see 2.4 and 4.2) in the non-reference dataset according to the reference dataset.

#2) This function returns a list, which contains the following objects (in order):

#2.1) Variants available only in the "reference" dataset.

#2.2) Variants available only in the "other" dataset.

#2.3) Variants available in both datasets that did not have the same allele pair.

#2.4) Correlation between EAFs before and after allele matching.

#2.5) Harmonised "reference" dataset.

#2.6) Harmonised "other" dataset.

#3) The function takes two data frames as arguments:

#3.1) reference: the dataset that will be used as the reference for allele matching.

#3.2) other: the dataset that will have its alleles flipped when they do not match with the corresponding alleles in the reference dataset.

#4) Formatting guidelines:

#4.1) Both datasets MUST have at least four columns:

#4.1.1) snp: contains a unique identifier of the genetic variant. This will normally be the rs number, but other naming conventions (e.g., chromosome:position, such as chr6:1094912) can be used. The naming convention MUST be consistent between datasets.

#4.1.2) a1: effect allele.

#4.1.3) a2: non-effect allele. Please ensure that the same naming conventions have been used in a1 and a2 (e.g, letter case, nomenclature of INDELs, etc).

#4.1.4) beta: corresponds to the effect (commonly a regression coefficient) of the variant on a given phenotype. If odds ratio are being used, they MUST be converted to ln(OR) before the function is applied.

#4.2) It is not essential to provide EAFs. However, if it is available, its column must be named 'eaf'.

#4.3) Additional columns (eg, chromosome, position, standard error, p-values, number of individuals and imputation quality) will remain unchanged.

harmonise_datasets <- function(reference, other) {

if(length(reference$snp)!=length(unique(reference$snp))) {

stop('The \'snp\' column in the reference dataset column contains non-unique values!')

}

if(length(other$snp)!=length(unique(other$snp))) {

stop('The \'snp\' column in the other dataset column contains non-unique values!')

}

reference$snp <- as.character(reference$snp)

reference$a1 <- as.character(reference$a1)

reference$a2 <- as.character(reference$a2)

other$snp <- as.character(other$snp)

other$a1 <- as.character(other$a1)

other$a2 <- as.character(other$a2)

#Identify SNPs not mutually available in both datasets

reference_only <- reference$snp[!reference$snp%in%other$snp]

other_only <- other$snp[!other$snp%in%reference$snp]

if(length(reference_only)==nrow(reference)) {

stop('The datasets do not have any variant in common!')

}

#Remove SNPs not commonly available in both datasets

#Sort both filtered datasets according to the snp column

reference <- reference[reference$snp%in%other$snp,]

reference <- reference[order(reference$snp),]

other <- other[other$snp%in%reference$snp,]

other <- other[order(other$snp),]

#Exclude SNPs that do not share allele pairs between data sets

snps_to_keep <- (reference$a1==other$a1 | reference$a1==other$a2) & (reference$a2==other$a1 | reference$a2==other$a2)

different_allele_pair <- reference$snp[!snps_to_keep]

reference <- reference[snps_to_keep,]

other <- other[snps_to_keep,]

#Now, flip alleles that do not match

flip_index <- reference$a1!=other$a1

other$a1[flip_index] <- reference$a1[flip_index]

other$a2[flip_index] <- reference$a2[flip_index]

other$beta[flip_index] <- -other$beta[flip_index]

#Compute correlation between EAFs before and after allele matching, and flip EAFs in the other datset

if('eaf'%in%colnames(reference) & 'eaf'%in%colnames(other)) {

eaf_cor <- cor(reference$eaf, other$eaf, use='c') #EAF correlation before allele matching

other$eaf[flip_index] <- 1-other$eaf[flip_index] #Flipping EAFs in the other dataset

eaf_cor <- c(eaf_cor, cor(reference$eaf, other$eaf, use='c')) #EAF correlation after allele matching

names(eaf_cor) <- c('before', 'after')

} else {

eaf_cor <- NA

}

return(list(reference_only=reference_only, other_only=other_only, different_allele_pair=different_allele_pair,

eaf_cor=eaf_cor,

reference=reference, other=other))

}
